# An α/β Hydrolase and Associated Per-ARNT-Sim Domain Comprise a Bipartite Sensing Module Coupled with Diverse Output Domains

**DOI:** 10.1371/journal.pone.0025418

**Published:** 2011-09-29

**Authors:** Eugene V. Nadezhdin, Margaret S. Brody, Chester W. Price

**Affiliations:** Department of Microbiology, University of California Davis, Davis, California, United States of America; Texas A&M University, United States of America

## Abstract

The RsbQ α/β hydrolase and RsbP serine phosphatase form a signaling pair required to activate the general stress factor σ^B^ of *Bacillus subtilis* in response to energy limitation. RsbP has a predicted N-terminal Per-ARNT-Sim (PAS) domain, a central coiled-coil, and a C-terminal protein phosphatase M (PPM) domain. Previous studies support a model in which RsbQ provides an activity needed for PAS to regulate the phosphatase domain via the coiled-coil. RsbQ and the PAS domain (RsbP-PAS) therefore appear to form a sensory module. Here we test this hypothesis using bioinformatic and genetic analysis. We found 45 RsbQ and RsbP-PAS homologues encoded by adjacent genes in diverse bacteria, with PAS and a predicted coiled-coil fused to one of three output domains: PPM phosphatase (Gram positive bacteria), histidine protein kinase (Gram negative bacteria), and diguanylate cyclase (both lineages). Multiple alignment of the RsbP-PAS homologues suggested nine residues that distinguish the class. Alanine substitutions at four of these conferred a null phenotype in *B. subtilis*, indicating their functional importance. The F55A null substitution lay in the Fα helix of an RsbP-PAS model. F55A inhibited interaction of RsbP with RsbQ in yeast two-hybrid and pull-down assays but did not significantly affect interaction of RsbP with itself. We propose that RsbQ directly contacts the PAS domains of an RsbP oligomer to provide the activating signal, which is propagated to the phosphatase domains via the coiled-coil. A similar mechanism would allow the RsbQ-PAS module to convey a common input signal to structurally diverse output domains, controlling a variety of physiological responses.

## Introduction

Organisms monitor an array of external and internal signals to adjust their behavior to changing circumstances. Signal transduction proteins that sense and convey these cues are often of modular design, with distinct input (sensory) and output (effector) domains; signal-dependent modulation of the interactions between these domains controls information flow to downstream targets [1]. The Per-ARNT-Sim (PAS) family represents a widely distributed sensory and interaction domain that can be adapted to a variety of signaling tasks [2]. Different PAS domains have only modest sequence identity but share a distinctive structural fold that can detect changes in chemical or physical parameters; this fold is characterized by five anti-parallel β strands flanked on one side by several α helices [3]. In some cases residues lining the interior of the fold directly interact with a small chemical ligand to trigger signaling [4], whereas in others an internally-bound cofactor such as FAD, FMN, or heme is needed to transduce fluctuations in redox, light energy, or gas concentration into a useful signal [5]-[8]. However, for a significant fraction of PAS domains a ligand or cofactor cannot be readily identified [3].

**Figure 1:**
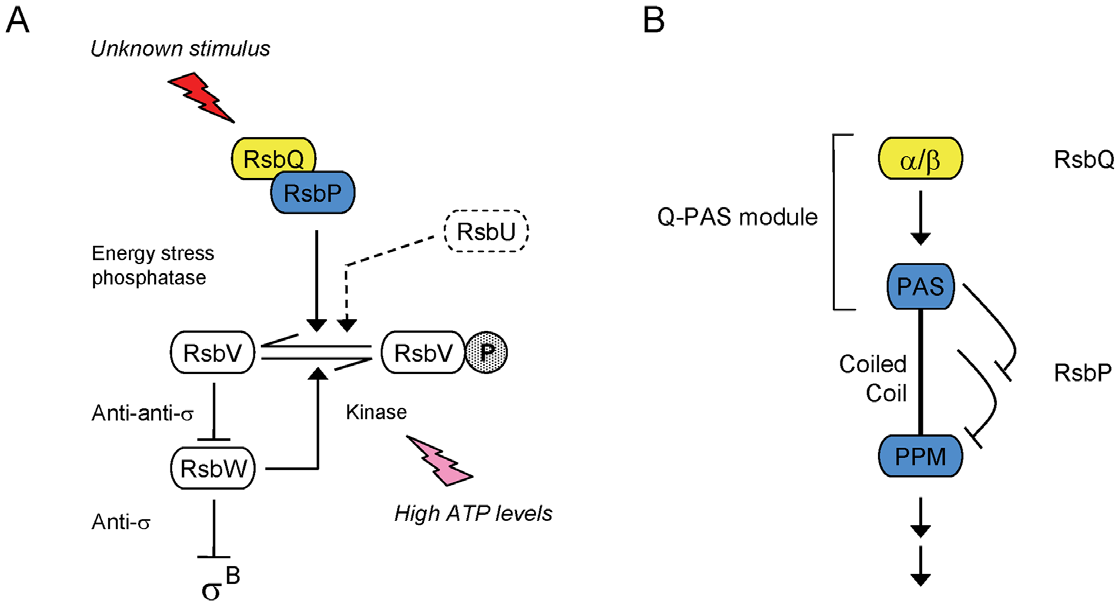
Signaling pathway that activates σ^B^ in response to energy stress. (A) Model of σ^B^ regulatory network [11], [12]. Separate signaling pathways converge on the RsbV anti-anti-σ and the RsbW anti-σ, the direct regulators of σ^B^. These pathways terminate with distinguishing serine phosphatases: RsbP (blue oval) and the RsbQ α/β hydrolase (yellow oval) are required for response to energy signals whereas RsbU (represented in dotted outline) is required for response to environmental signals. In unstressed cells RsbW phosphorylates and inactivates its RsbV antagonist, allowing RsbW to sequester σ^B^ in an inactive complex. During the stress response RsbV-P is dephosphorylated, allowing it to bind RsbW and force the release of σ^B^. Horizontal arrows show the conversion between RsbV and RsbV-P (with phosphate as stippled P). Full arrowheads indicate positive action and T-headed arrows inhibiting action. The signal that activates the RsbP phosphatase is unknown (red stimulus). However, during energy stress RsbV-P dephosphorylation is promoted by a decrease in cellular ATP levels (magenta stimulus) and an accompanying decrease in RsbW kinase activity. (B) Mechanism of RsbP phosphatase activation [13]. In this model, a central coiled-coil holds the C-terminal PPM phosphatase domain (blue PPM oval) inactive. In the presence of signal the N-terminal PAS domain (blue PAS oval) counters the negative effect of the coiled-coil, activating the phosphatase and inducing σ^B^. The catalytic triad of the RsbQ hydrolase (yellow α/β oval) is also required for activation, suggesting that RsbQ and the PAS domain together comprise a sensory module.

Understanding the means by which different PAS domains sense and communicate diverse signals is necessary to identify common themes. Here we use a bioinformatic and genetic approach to investigate a bacterial sensing module that consists of two parts: an α/β hydrolase and a separate PAS domain that is covalently attached to structurally diverse output domains in different organisms. As shown in Fig. 1A, the prototype for this module is found in the RsbQ-RsbP signaling pathway of the Gram positive bacterium *Bacillus subtilis* [9], [10], where both the RsbQ hydrolase and RsbP serine phosphatase are normally required to activate the general stress transcription factor σ^B^ in response to energy limitation [11], [12].

The structural genes for RsbQ and RsbP lie in the bicistronic *rsbQP* operon [10]. Based on sequence analysis [13], the RsbP product has an N-terminal PAS domain (RsbP-PAS), a central helical region with potential to form a parallel coiled-coil, and a C-terminal PPM serine phosphatase domain, sometimes called a PP2C domain [14], [15] after the defining member of the family. As shown in Fig. 1B, genetic analysis indicates that RsbP-PAS has a positive role in controlling phosphatase activity, acting by countering the negative role of the central region [13]. The RsbQ hydrolase also has a positive role that requires the integrity of its catalytic triad, implying it provides an activity needed for RsbP function [9]. This activity is thought to produce a small molecule that binds within the RsbP-PAS fold [16], but as yet there is no experimental support for this hypothesis. No ligand or cofactor has been found associated with RsbP-PAS, and the signal that controls RsbP remains unknown.

During our initial BLAST searches to explore the distribution of domains related to RsbP-PAS, we noticed that its closest homologues were encoded in tandem with a clear RsbQ homologue, as is the case in *B. subtilis*. However, the predicted output domains covalently associated with these RsbP-PAS homologues included not only PPM phosphatases, but also histidine protein kinase and diguanylate cyclase domains. We report here the results of a PSI-BLAST search that found 45 such PAS-containing proteins, and a multiple alignment that suggested residues representative of the class. Genetic analysis indicated that some of these residues were essential for RsbP-PAS function in *B. subtilis*, and that one was important for direct interaction with RsbQ.

## Results

### Distribution of the RsbQ-PAS module

**Figure 2:**
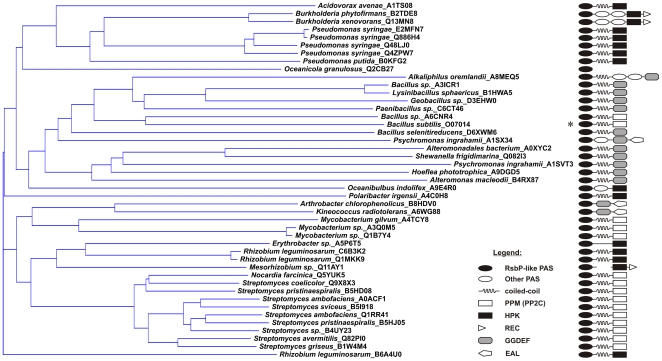
Relationship among RsbP-PAS-like domains and architecture of their parent proteins. Left, neighbor-joining tree representing 45 RsbP PAS homologues that form Q-PAS modules (see Methods). PAS-containing proteins are identified by species name and UniProt identifier [18]. Right, domain architecture of each protein determined using the Conserved Domain Database [19], with *B. subtilis* RsbP indicated by the asterisk. The RsbP-PAS like domain (filled oval) is always the most N-terminal in the parent protein and a homologue of the RsbQ α/β hydrolase (not shown) is encoded immediately upstream. Five proteins have additional PAS domains (open oval) not closely related to RsbP-PAS. Three major classes of output domains are shown: PPM phosphatase (open rectangle), histidine protein kinase (closed rectangle), and diguanylate cyclase (grey octagon). Three of the kinases have a C-terminal response regulator domain (open triangle) and three of the cyclases have a C-terminal phosphodiesterase domain (open pentangle). For two PAS homologues the output domain is uncertain: *O. granulosus* PAS (Q2CB27) lies at the end of a contig, whereas *Mesorhizobium* sp. PAS (Q11AY1) is separated from a histidine kinase domain by frame shift. Potential of the central helical regions to form coiled-coils was assessed using the PCOIL program (see Methods).

We used a PSI-BLAST [17] search to identify homologues of the RsbP-PAS domain encoded downstream from an RsbQ homologue, in the same configuration as *B. subtilis rsbQP*. Beginning with a query of RsbP residues 1-109 [13], we chose from the first iteration all RsbP-PAS homologues partnered with RsbQ, then used these in a second iteration. This yielded 44 RsbP-PAS homologues, all of which were significantly similar to the query (e value less than 3e–18). Each of the 44 that were numerically below this cut-off had an RsbQ-like companion, which was absent from virtually all those above it. The relationship among these 44 RsbP-PAS homologues is shown in Fig. 2, labeled by organism and the UniProt identifier of the parent protein [18]. One homologue (*Alteromonas macleodii*_B4RX87) from above the cut-off did have an RsbQ partner and was included to broaden sequence diversity. For convenience we refer to all 45 RsbP-PAS-like domains as RsbP-PAS.

We catalogued the domain architectures of the 45 proteins containing an RsbP-PAS homologue, based on profiles available in the Conserved Domain Database [19]. Three themes are apparent, as shown in Fig. 2. First, even in proteins with multiple input domains, RsbP-PAS is the most N-terminal. Thus it is always encoded directly adjacent to the RsbQ homologue (not included in Fig. 2). Second, RsbP-PAS is associated with three different output domains: PPM phosphatase in Gram positive bacteria; histidine protein kinase (or hybrid kinase) in Gram negative bacteria; and diguanylate cyclase (GGDEF) in both lineages. Third, in 36 of the 45 proteins, RsbP-PAS is connected to the output domain by a potential coiled-coil region of at least 21 residues. In only one case is the predicted coiled-coil absent from a protein with single input and output domains (*Erythrobacter* sp_A5P6T5). Other exceptions lacking the coiled-coil region include proteins in which one or two additional PAS domains lie C-terminal to RsbP-PAS; others that have an additional phosphodiesterase domain (EAL) C-terminal from a cyclase domain; and two that have no associated output domain, either because sequencing is incomplete (*Oceanicola granulosus*_Q2CB27) or possibly in error (*Mesorhizobium* sp_Q11AY1).

We note that three of the organisms included in Fig. 2 encode two separate copies of RsbQ-PAS. In *Streptomyces ambofaciens* and *S. pristiniaspiralis*, both copies are associated with phosphatase output domains. In *Psychromonas ingrahamii*, both copies apparently regulate cyclic di-GMP metabolism. The PAS domain of one (A1SVT3) is linked to a diguanylate cyclase domain via a predicted coiled-coil, whereas that of the other (A1SX34) is linked to tandem cyclase and phosphodiesterase domains via a heterologous PAS domain. Based on the bioinformatic analysis, we conclude that RsbQ and RsbP-PAS form a bipartite input module that regulates the activity of at least three structurally different output domains.

### Multiple alignment suggested residues characteristic of RsbP-PAS

We constructed a ClustalW alignment [20] of 24 representative RsbP-PAS sequences that have an adjacent RsbQ partner. We then compared the RsbP-PAS alignment with a similar alignment of 24 close homologues that have no neighboring RsbQ. This allowed us to eliminate from consideration residues shared with these close homologues as well as residues conserved among PAS domains in general. We expected the remaining differences would reflect residues specific to RsbP-PAS homologues in close association with RsbQ.

**Figure 3:**
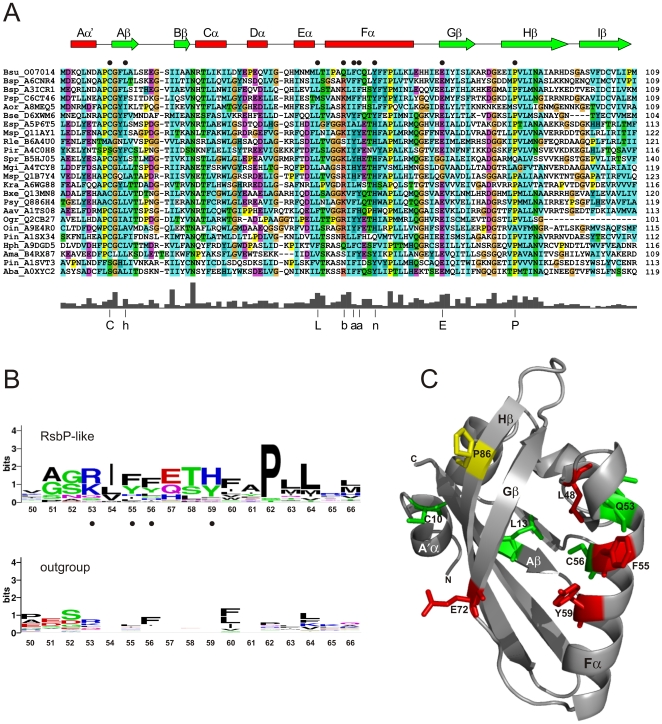
Distinctive residues in RsbP-PAS-like domains. (A) Multiple sequence alignment of the 24 most diverse RsbP-PAS like domains from Fig. 2, labeled by genus-species abbreviation and UniProt identifier. Numbers on the right indicate position of the terminal residue of the domain within each protein. Color scheme is standard ClustalW: blue for WLVIMFAC; cyan for HY; green for TSNQ; magenta for DE; red for KR; orange for G; and yellow for P. Predicted secondary structural elements of *B. subtilis* RsbP-PAS (from Fig. 3C) are shown as a cartoon above the alignment, with α helices in red and β strands in green. Black circles indicate the locations of nine RsbP residues analyzed here by alanine substitution (see text). The plot below the alignment shows average sequence conservation, with the 75% consensus at the nine positions indicated by C, L, E or P in one-letter code, or h for hydrophobic (ACFILMVW), b for basic (KR), a for aromatic (FHWY), n for neutral aromatic (HY). (B) Sequence conservation within predicted Fα helices of the 24 RsbP-PAS homologues from panel A (*top logo*) and the corresponding region of the comparison outgroup (*bottom logo*). Logos represent relative frequency and information content at each position, numbered according to the *B. subtilis* RsbP-PAS sequence. Black circles denote RsbP residues chosen for genetic analysis. (C) Ribbon diagram depicting a computation model of the *B. subtilis* RsbP-PAS domain. Key secondary elements are labeled according to standard PAS nomenclature [3]. Positions of the nine residues probed by alanine substitution are represented by color-coded sticks that denote their effects on RsbP function: green for no significant effect; yellow for substantial loss of function; red for complete loss of function (null phenotype). Experimental data for the alanine substitutions is shown in Fig. 4.

To make the RsbP-PAS alignment, we used the HHfilter utility from the MPI Bioinformatics Toolkit [21] to select the 24 most diverse sequences from those shown in Fig. 2. Each of the 24 shared no more than 50-55% identity with any other member of the set, reducing bias in the ClustalW alignment shown in Fig. 3A. For the comparison outgroup, the same PSI-BLAST search that yielded the 45 RsbP-PAS homologues shown in Fig. 2 also provided 45 related PAS homologues that lacked an RsbQ homologue encoded upstream. The 24 most diverse of these produced the contrasting ClustalW alignment shown in Fig. S1.

**Figure 4:**
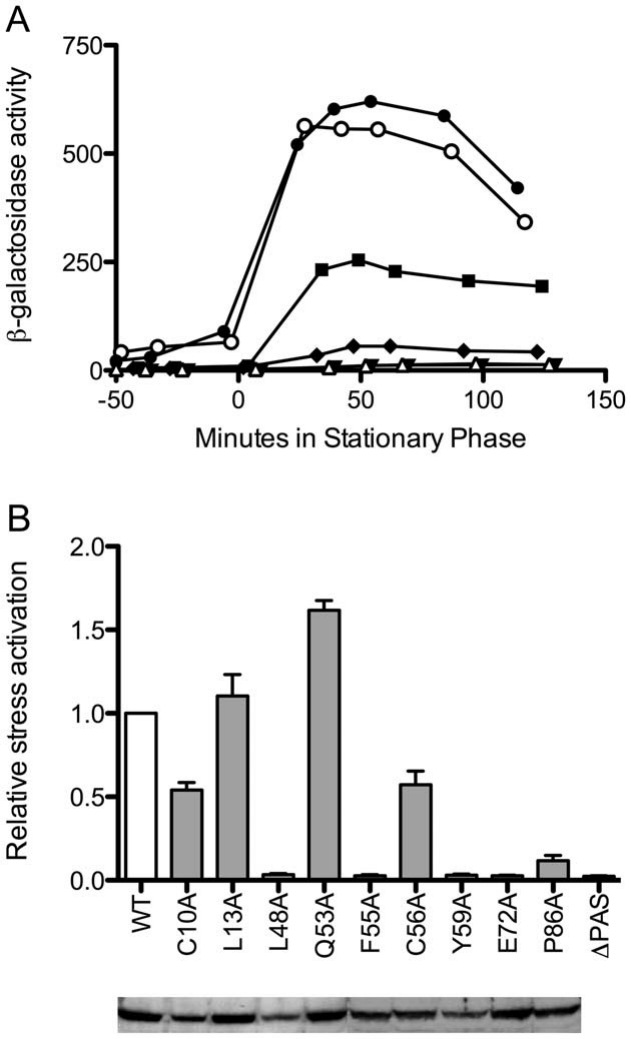
Effects of RsbP-PAS substitutions on σ^B^ induction in response to energy stress. (A) Effects of representative substitutions on σ^B^ activity, measured using a *ctc-lacZ* reporter fusion. Open circles indicate β-galactosidase accumulation in wild type positive control (PB198); open triangles, ΔPAS negative control (PB715); filled squares, C10A (PB1058); filled circles L13A (PB1059); filled inverted triangles, F55A (PB1062); filled diamonds, P86A (PB1067). Time 0 indicates entry into stationary phase. (B) *Upper*, relative activation following energy stress, with white bar showing the wild type control (WT; PB198) taken as 1. Activation is defined as maximum activity in response to stress; average activation for WT was 400 units. Grey bars indicate congenic strains bearing RsbP-PAS substitutions or a deletion: C10A (PB1058); L13A (PB1059); L48A (PB1060); Q53A (PB1061); F55A (PB1062); C56A (PB1063); Y59A (PB1064); E72A (PB1065); P86A (PB1067); ΔPAS (PB715). Error bars indicate SEM of at least two independent assays. *Lower*, Western blot of extracts from the same strains (100 µg protein each) separated by PAGE and probed with anti-RsbP antibody.

Comparison of the two multiple alignments indicated their close relationship. However, there were two striking differences. First, the outgroup sequences usually terminated with the widespread DIT motif identified by Möglich *et al*. [3], [22], [23]; this motif is thought to be important for coupling PAS output to an adjacent coiled-coil or amphipathic linker. By contrast, none of the RsbP-PAS-like sequences had the DIT motif (see Fig. 3A), suggesting a different means of pairing PAS with downstream elements. Second, the sequence of the predicted Fα helix of the RsbP-PAS homologues was notably conserved relative to the corresponding region of the outgroup; this distinction is represented by the logos [24] shown in Fig. 3B.

These Fα differences are not altogether surprising because Eα, Fα and the FG loop represent the most diverse elements of the PAS fold [3]. In some PAS domains this diversity contributes to the binding of specific ligands within the hydrophobic pocket formed by the β sheet and the helical regions, and in others it contributes to critical protein-protein interactions. The consistency of the Fα alignment in Fig. 3A is therefore telling, and allowed us to choose four residues from *B. subtilis* RsbP for further analysis: Q53, F55, C56, and Y59. Another nearby residue, P62, is highly conserved among RsbP-PAS homologues. However, in the absence of an experimentally determined structure, it is not clear whether this proline represents an unusual but key residue within Fα or acts instead as a helix-breaker. We therefore relied on the other four residues to represent Fα.

Further inspection suggested five additional residues for analysis (Fig. 3A). For example, in both alignments an interesting hPhG motif was apparent between the predicted A'α flanking helix and the Aβ strand of RsbP-PAS, where h designates a hydrophobic residue. However, cysteine occupied the second h position in 80% of the RsbP-PAS-like sequences and in only a third of the outgroup. This and similar considerations allowed us to chose C10, L13, L48, E72, and P86 from *B. subtilis* RsbP. In total, nine representative residues were selected, and these were scattered throughout the predicted PAS structure in the A'A loop, Aβ, the EF loop, Fα, Gβ and Hβ.

Preliminary crystallization of the RsbP-PAS domain has been reported [25] but the structure is not yet available. Therefore, the secondary structure assignments included in Fig. 3A were based on the computation model shown in Fig. 3C. This model was constructed by the i-tasser server [26], which threaded the *B. subtilis* RsbP-PAS sequence against the four structures chosen by the server as the best templates (see Materials and Methods). The model has the canonical PAS fold of five anti-parallel β strands flanked on one side by four α helices; the confidence score of 0.80 suggests an RMSD of 2.6 (+/- 1.9) Å, relative to the true structure. The anti-parallel β strands are highly conserved among known PAS structures [3], and this contributes to the favorable confidence score of the model. By contrast, the helices are more variable, and their length and position may differ in the actual structure. Although this computational model is not equivalent to an experimentally determined structure, it is helpful in assessing key features. For example, of the residues chosen from inspection of the multiple alignments, Q53 and C56 lie on one face of the Fα helix whereas F55 and Y59 lie on the opposite face (Fig. 3C). Should the true register of the helix differ by as much as half a turn, these four residues together still sample both the interior of the fold as well as an exterior surface that is potentially important for protein-protein interaction.

### Signaling phenotypes elicited by directed substitutions in *B. subtilis* RsbP-PAS

We made missense mutations in *B. subtilis rsbP* that introduced alanine substitutions at each of the nine residues of interest, then incorporated these at the chromosomal *rsbP* locus by a single crossover event (see Materials and Methods). Expression of a *lacZ* reporter showed the effect of each substitution on σ^B^ activity as cells were subjected to energy depletion at the end of exponential growth. A typical assay is shown in Fig. 4A. Here a strain bearing the L13A substitution manifested a response similar to the wild type control; C10A and P86A conferred reduced responses, and a strain bearing the F55A substitution was unable to activate σ^B^ at all, with a response similar to the negative control.

Fig 4B summarizes the results of multiple assays for each of the nine substitutions, expressed relative to wild type. The substitutions fell into two functional categories. The first contained C10A, L13A, Q53A and C56A, which had only modest effects, conferring responses ranging from about 50 to 150% of the wild type control. The second included P86A, which had a weak but detectable response, and L48A, F55A, Y59A and E72A, which elicited complete loss-of-function (null) phenotypes, with responses similar to the negative control. For this second category in particular, we could not be certain that the substituted proteins retained their native structures. However, under the growth conditions in which their function is normally manifest, all but three of the mutants had steady-state protein levels at least 80% of wild type (Fig. 4B). The three exceptions were C10A, L48A, and Y59A, all of which had steady-state levels between 50 and 60% of wild type. These results indicate that each of the nine mutant proteins had about the same intracellular stability as wild type RsbP, suggesting that their structures were largely unaffected by the substitutions. The results further indicate that reduced levels of mutant RsbP may have contributed to the partial phenotype elicited by C10A, and to the null phenotypes caused by L48A and Y59A.

The mild phenotypes conferred by the C10A and C56A single substitutions support two conclusions. First, cysteine residues are integral to well-characterized mechanisms of oxidative stress sensing [27]. Our results show that neither the C10 nor the C56 side chain was significant for RsbP function, at least under the assay conditions used here. Second, the position of C56 corresponds to the H77 or the H194 residue that coordinates heme binding within the PAS folds of *E. coli* Dos [7], [8] or *Sinorhizobium meliloti* FixL [28], respectively. Although most of the RsbP-PAS sequences shown in Fig. 3A have a hydrophobic or neutral aromatic residue at this position, two have a histidine and two more (including *B. subtilis* RsbP) a cysteine, which binds heme in some molecular contexts. Notably, loss of the C56 side chain was not critical for function in our standard assay. By contrast, the side chains of L48, F55, Y59 and E72 were required, and side chain of P86 was an important contributor. We therefore focused on this second group of substitutions for the remainder of our study.

### The F55A substitution differentially affected RsbP interactions

**Figure 5:**
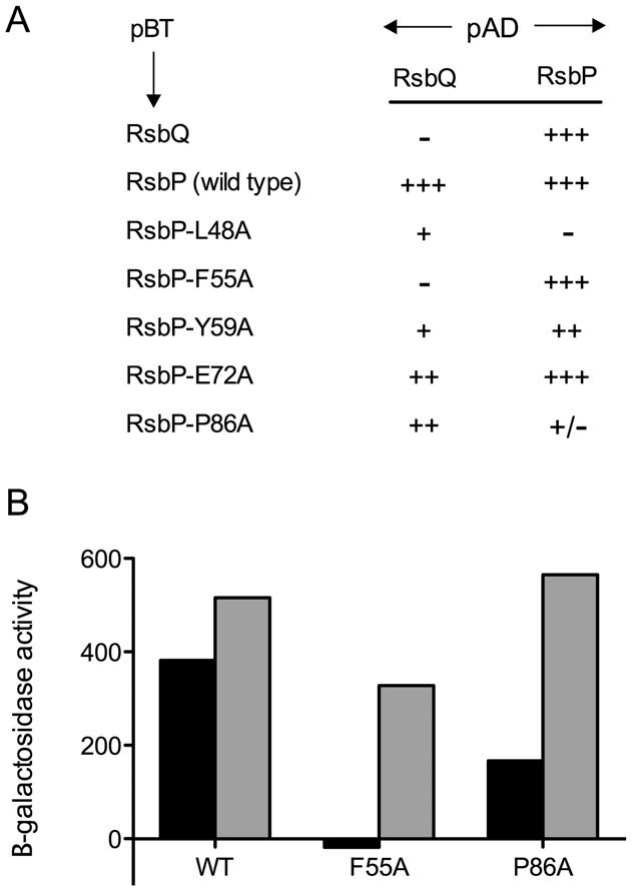
Effects of RsbP-PAS substitutions on protein interactions in the yeast two-hybrid system. (A) Colony lift screen of indicated full-length proteins fused to the GAL4 DNA binding domain in pGBT9 (pBT) or the GAL4 activation domain in pGAD424 (pAD). Double transformants of yeast strain SFY526 were grown on selective medium and treated with X-Gal, as described in Methods. Observed β-galactosidase activities are indicated as +++ (strong), ++ (medium) or + (weak) blue color; +/-, very weak blue color; or –, no blue color. (B) Quantitation of key interactions, expressed as β-galactosidase accumulation in liquid-grown cultures. Wild type RsbP (WT), RsbP-F55A or RsbP-P86A was fused to the GAL4 DNA binding domain in pGBT9 and paired with either wild type RsbQ (black bars) or wild type RsbP (grey bars) fused to the GAL4 activation domain in pGAD424. Activities shown are the average of four independent double transformants grown in selective medium, then sampled during logarithmic growth in rich medium; these have been corrected by subtracting the average activity of each GAL4 DNA binding construct expressed alone (self-activation control).

Because PAS domains frequently modulate protein-protein interactions, we tested the five most deleterious substitutions for possible phenotypes in the yeast two-hybrid system. An earlier study found that RsbP appeared to interact with RsbQ in yeast [9]. Here we confirm and extend these earlier results, showing that wild type RsbP strongly activated transcription of the reporter fusion when paired with either RsbQ or with itself (Fig. 5A). This strong self-self activation suggested that RsbP forms a homo-oligomer.

Based on the plate lift assays summarized in Fig. 5A, the mutant phenotypes fell into four classes, two of which appeared to differentially affect interaction of RsbP with its partners. Class One substitutions (F55A and Y59A) significantly reduced interaction with RsbQ while leaving the interaction with wild type RsbP largely unaffected, whereas Class Two (P86A) appeared to have the opposite phenotype. Class Three (L48A) impeded interaction with both binding partners. Lastly, Class Four (E72A) did not significantly affect either interaction, and therefore may alter another critical but presently unknown aspect of PAS function. Fig. 5A includes only results obtained with the mutant forms of RsbP expressed in the pBT binding domain; reciprocal assays (with the mutant forms expressed in the pAD activating domain) were essentially the same. Results were equally unaffected whether the self-self assay of RsbP was done between a given mutant form and wild type (Fig. 5A), or between a given mutant form and itself (data not shown).

The plate lift assays shown in Fig. 5A are convenient for screening but are subject to false positive or negative artifacts [29]. We therefore used a more quantitative liquid assay to test the phenotypes of two representative substitutions, which appeared to differentially affect the interaction of RsbP with RsbQ (F55A), and RsbP with itself (P86A). As shown in Fig 5B, F55A indeed eliminated activation of the reporter fusion when paired with RsbQ while leaving self-self activation largely unaffected. The ability to test the effect of F55A with two different partners lent confidence to the specificity observed: retention of the self-self activation indicated that the F55A protein was stably expressed, non-toxic, and capable of interacting in the yeast two-hybrid system. Thus for F55A the quantitative assay was consistent with the differential interaction suggested by the plate lift assay. By contrast, the P86A substitution had little effect on activation with either partner in the quantitative assay, and was not studied further.

### Biochemical analysis confirmed the interaction phenotype of F55A

**Figure 6:**
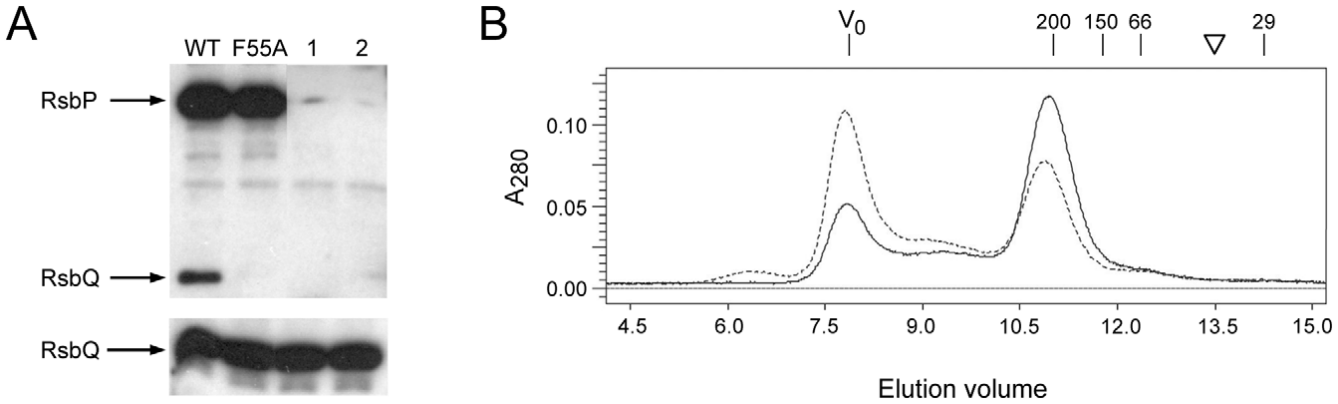
The RsbP-F55A substitution inhibits interaction with RsbQ in vitro but does not affect RsbP oligomerization. (A) Pull-down assays using His-tagged wild type or F55A RsbP protein attached to magnetic beads. RsbP-coated beads were incubated with extracts from wild type *B. subtilis* and washed with buffer containing 20 mM imidazole to remove unbound proteins. Bound proteins were then eluted from the beads with buffer containing 250 mM imidazole. Wash and elution fractions were separated by SDS-PAGE and probed with anti-RsbQ antibody, which also cross-reacted with His-tagged RsbP. *Upper*, His-tagged RsbP and native RsbQ bound to beads and eluted with high imidazole; *lower*, one-quarter of the amount of unbound RsbQ eluted in the low imidazole wash. WT and F55A indicate species of His-tagged RsbP bound to the beads; control lane 1, reaction using beads coated with proteins from a parallel purification from *E. coli* cells not expressing His-tagged RsbP; control lane 2, reaction using uncoated beads. (B) Gel filtration elution profile of His-tagged RsbP wild type or F55A mutant protein indicates a hydrodynamic radius corresponding to a spherical particle with a mass of 200 kDa. Profile shows A_280_ of the wild type (solid) or F55A mutant (dashed) form on the ordinate and cumulative elution volume on the abscissa. V_0_ indicates void volume of the Superose 12 column (7.9 ml); numbers indicate elution of standard proteins β-amylase (200 kDa; 11.2 ml), alcohol dehydrogenase (150 kDa; 11.8 ml), BSA (66 kDa; 12.4 ml) and carbonic anhydrase (29 kDa; 14.3 ml). Inverted triangle indicates elution position expected for a spherical particle with the mass of His-tagged RsbP monomer (48.3 kDa; 13.5 ml). SDS-PAGE gel analysis (not shown) found absorbance at V_0_ was due to an RsbP aggregate.

The differential effects of the F55A substitution were also investigated using purified RsbP proteins. We first tested the RsbQ-RsbP interaction using pull-down assays, attaching His-tagged, wild type RsbP to Ni-NTA magnetic agarose beads. However, RsbP was unable to form detectable complexes with purified RsbQ in this assay (data not shown). This is contrary to the results of the yeast two-hybrid assay, and suggested that the intracellular environment might contain a factor important for the interaction. We developed a cell extract model to test this notion. Significantly, His-tagged wild type RsbP attached to Ni-NTA magnetic beads effectively pulled down native RsbQ from *B. subtilis* cell extracts, but RsbP-F55A beads were unable to complex RsbQ under these conditions (Fig. 6A). These results indicate that some feature of the extract which facilitates the RsbQ-RsbP interaction is transferable to the in vitro system, and that the F55A substitution inhibits this interaction.

To test the RsbP-RsbP interaction, we analyzed purified wild and mutant RsbP proteins on a Superose 12 size exclusion column (Fig. 6B). The wild-type elution profile provided evidence of a stable RsbP homo-oligomer, indicated by a hydrodynamic radius consistent with a spherical 200 kDa particle. Based on a predicted monomer mass of 48,325 for His-tagged RsbP, this result suggested that the bulk of the preparation formed a tetramer. Inspection of the elution profile indicated that some RsbP appeared as a large aggregate in the void volume, whereas none eluted at the position expected for a monomer. The elution profile of the F55A form was essentially the same as wild type, with a greater aggregate fraction (Fig. 6B). Because the F55A form and the wild type protein were equally stable, both in vivo (Fig 4B) and in vitro (data not shown), this greater aggregate fraction may indicate a small effect of F55A on RsbP oligomerization. Thus two biochemical assays confirmed the results from the yeast two-hybrid system: the F55A substitution within the PAS domain had at most only modest influence on the ability of RsbP to form a homo-oligomer, but significantly affected the interaction of RsbP with RsbQ.

### The PAS domain was both necessary and sufficient for the interaction of RsbP with RsbQ

The behavior of the F55A substitution in yeast two-hybrid as well as pull-down assays indicated that the PAS domain was important for the interaction of full-length RsbP with RsbQ. Additional assays in the yeast two-hybrid system estimated the contribution of the different regions of RsbP to this interaction. Based on the data shown in Table 1, the PAS domain alone still interacted with RsbQ, whereas the predicted coiled-coil region and PPM phosphatase domain did not, either alone or in combination. We interpret these data to indicate that the PAS domain provides determinants essential for RsbQ to effectively bind RsbP. This interpretation is in accord with the consistent genomic context of RsbQ homologues and the RsbP-PAS domain, which is in turn found in signaling molecules that vary both with respect to the presence of an adjacent coiled-coil and the type of output domain (Fig. 2).

**Table 1:** Yeast two-hybrid interactions of RsbQ with different domains of RsbP^a^.

	Region in pAD activating domain^b^			
	Full RsbP	PAS	Coil-PPM	PPM
RsbQ in pBT binding domain	+++	+	–	–

a +++ → +, strong to weak blue color in colony lift assay; –, no blue color detected.

b Full RsbP  =  residues 1–403; PAS = 1–111; Coil-PPM = 110–403; PPM = 167–403.

## Discussion

We used a bioinformatic analysis to identify a bipartite sensing module implicated in controlling three different output domains in diverse bacteria: PPM phosphatase, histidine protein kinase, and diguanylate cyclase. These output domains are commonly found in signal transduction pathways that control response to the local environment and thus contribute to bacterial adaptation and survival. Our genetic analysis of *B. subtilis* RsbQP, the prototype signal transduction pathway employing this module, bears on the mechanism by which the RsbQ α/β hydrolase and the associated RsbP-PAS domain sense an activating signal.

**Figure 7:**
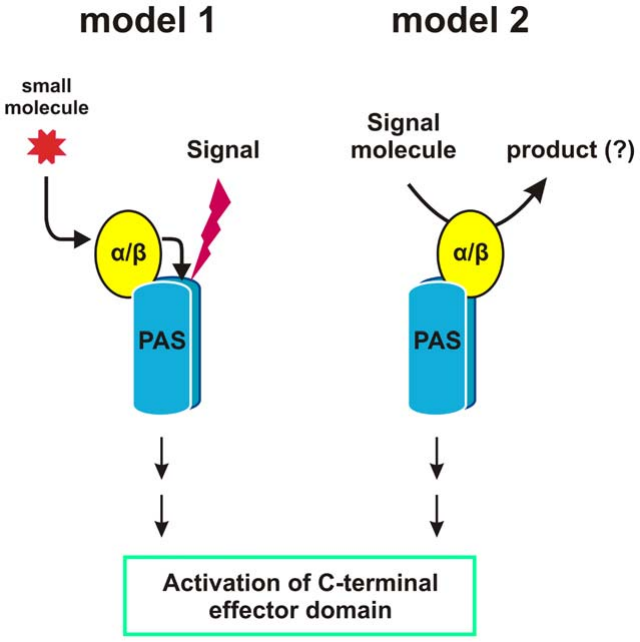
Alternative models of signal sensing by the RsbQ-PAS module. Both models entail direct interaction between the RsbQ α/β hydrolase and the PAS domain of an oligomeric signaling protein. This interaction generates a structural change that is communicated to the effector domains. In Model 1, the α/β hydrolase (yellow) converts a small molecule to a product it delivers to the interior of the PAS fold (blue). This product can serve as the direct activating signal, or can confer upon the PAS domain the ability to detect the signal, as would be the case for a gas, redox or light-detecting ligand. In Model 2, the α/β hydrolase forms a long-lived association with the PAS domain. Entry of the signal molecule into the active site of the hydrolase induces a change in its interaction with PAS. In this model, conversion of the signal molecule to product is not strictly necessary but may serve as a timing mechanism.

Previous work found that substitutions at residues comprising the catalytic triad of the RsbQ hydrolase conferred a null phenotype, indicating that integrity of the active site was required for signaling [9]. Three lines of evidence from the present study suggest that direct contact between RsbQ and the PAS domain of RsbP is also necessary. First, the RsbP-PAS domain (and not the potential coiled-coil or phosphatase domain) was both necessary and sufficient for interaction with RsbQ in the yeast two-hybrid system. Second, full-length RsbP and RsbQ interacted in both yeast two-hybrid and pull down assays, and this interaction was specifically disrupted by the F55A substitution in the predicted Fα helix of RsbP-PAS. F55A had little effect on the ability of RsbP to interact with itself in the two-hybrid system or to form a homo-oligomer in vitro, nor did it affect RsbP levels in vivo. Third, we infer that RsbQ interacted with RsbP-PAS in *B. subtilis*. This inference is based on the finding that the F55A substitution, which disrupted the RsbQ interaction in vitro, conferred a null signaling phenotype in vivo. These results can be understood in terms of two models in which direct contact between the α/β hydrolase and the PAS domain is required for signal transduction (Fig. 7). The models differ with respect to which partner – the hydrolase or the PAS domain -- is the primary signal sensor.

Model 1 is an extension of the earlier proposals of Brody *et al*. [9] and Kaneko *et al*. [16]. In this model, the α/β hydrolase converts a small molecule to a product that is itself the activating signal, or is an accessory sensor molecule -- analogous to heme in *E. coli* Dos -- required for the PAS domain to detect the activating signal (Fig. 7, *left*). In either case, we now suggest that the hydrolase must deliver this product to the hydrophobic cleft of the PAS fold by direct protein-protein interaction. In this model, the catalytic activity of the hydrolase is essential but PAS is the primary sensing domain.

By contrast, in Model 2 the α/β hydrolase is the sensor. Here the activating molecule binds within the catalytic site of the hydrolase, and this occupancy is signaled to the PAS domain by direct contact (Fig. 7, *right*). The LuxPQ quorum sensing system of *Vibrio harveyii* provides one well-characterized example in which bacterial PAS domains serve as sites of interaction with a separate sensing protein [30]. In this system, the LuxP binding protein forms a complex with the two periplasmic PAS domains of the LuxQ hybrid kinase. Association of the small autoinducer-2 molecule with LuxP triggers changes in the quaternary structure of the complex, which in turn leads to changes in the signaling output of the system. Our Model 2 proposes that a similar mechanism underpins the RsbQ-PAS cytoplasmic sensing module we identified. In this model, integrity of the hydrolase active site may be required only for signal molecule binding, and its catalytic activity may not be necessary. However, subsequent catalysis could provide a timing mechanism to prevent continued signaling.

How might the RsbQ α/β hydrolase interact with RsbP-PAS? Intra- and intermolecular interactions of PAS domains are commonly mediated by the five-stranded anti-parallel β sheet, which is the most highly conserved region of the domain, and may also involve pathway-specific N- or C-terminal helices that flank the β2-α4-β3 core structure [3]. However, some interactions of bacterial PAS domains engage the Fα helix or the FG loop [31], [32]. Notably, among RsbP-PAS-like domains associated with an RsbQ homologue (Fig. 2), the Fα helix is highly conserved (Figs. 3A and B). One explanation for this conservation is that Fα frequently contains residues important for coordinating ligand binding within the hydrophobic cleft of the fold [3], and all RsbP-PAS domains might bind a similar ligand. However, another explanation is that Fα might provide critical determinants for RsbQ binding, either to deliver a ligand (model 1) or to communicate the activating signal (model 2). This latter explanation is consistent with the null phenotypes conferred by alanine substitutions at the conserved F55 and Y59 positions of RsbP-PAS, both of which also disrupt interaction with RsbQ in the yeast two-hybrid system (Figs. 4 and 5). If the computation model shown in Fig. 3C is substantially correct, F55 and Y59 lie on the outer face of Fα, available for intermolecular contact. By contrast, Q53 and C56 lie on the opposite face of Fα and appear to comprise one boundary of the hydrophobic cleft; alanine substitutions at these residues had no significant effect on RsbP signaling.

How might RsbP-PAS communicate with structurally diverse output domains? Möglich *et al*. [3] proposed a general model for PAS signaling that relies on changes in the quaternary structure or dynamics of an oligomeric protein. They further proposed that these changes are conveyed in part by helices or coiled-coils that essentially act as rigid levers to couple the PAS and output domains. This model is attractive because it eliminates the need to invoke adaptive sequence changes to accommodate tertiary contact between a given PAS domain and the different output domains it might control. Experimental support for the model comes from hybrid proteins that replaced one PAS domain with another by a splice within the coiled-coil, thereby placing the output domain under control of a different input signal [22].

The results of our study of *B. subtilis* RsbP are consistent with the general model of PAS interdomain signaling of Möglich *et al*. [3]. Based on genetic analysis, Brody *et al*. [13] proposed the model of domain communication within RsbP shown in Fig. 1B, in which RsbP-PAS regulates the PPM phosphatase domain via the central α-helical region. From the conservation of this domain organization among the homologues shown in Fig. 2 we infer that RsbP-PAS employs a similar mechanism to regulate the three different output domains with which it is commonly associated. Two of the domains regulated by RsbP-PAS -- histidine protein kinase and diguanylate cyclase -- are known to function as dimers [33], [34]. However, less is known regarding the state of bacterial phosphatases like RsbP, which belong to PPM Subfamily II [13]-[15]. The gel filtration results presented here indicate that RsbP forms a stable oligomer in solution. Thus all three output domains regulated by RsbP-PAS are found in oligomeric proteins, and the central helical regions shown in Fig. 2 could form parallel coiled-coils, as predicted by the pcoil program, and in accord with the general model [3].

The physiological cue that initiates signal transduction via the RsbQ-PAS module remains mysterious. We note that the 45 examples shown in Fig. 2 are found in organo-heterotrophs that typically inhabit a soil or water environment, or are associated with plants. Many are able to switch between different forms of existence that promote growth and survival. All are capable of aerobic respiration. However, only in *B. subtilis* is the regulatory target of the module known. Because the RsbP phosphatase is normally required for activation of the general stress factor σ^B^ in response to energy depletion [10], it has been widely assumed that the RsbQ-PAS module senses energy status of the cell. However, there is no experimental support for this assumption and it may be gratuitous, as a signal of declining energy levels may also enter the *B. subtilis* network via the RsbW kinase, whose Km for ATP is in the physiological range [35], [36]. RsbP is more active than the RsbU environmental phosphatase in cells growing logarithmically under standard laboratory conditions [37], and thus sets the basal level of σ^B^ activity by countering the activity of the RsbW kinase (Fig. 1A). RsbP may therefore respond to a signal other than energy stress in order to adjust this basal level under different growth conditions, as previously proposed [10], [13].

Two studies have shown that the RsbP phosphatase is required for the energy branch of the σ^B^ network to respond to (i) a decrease in ATP levels [38] or (ii) an increase in red light illumination [39], and these signals are important for bacterial survival in the environment. However, both studies monitored output of the full signaling network and did not establish that RsbP directly sensed the ATP or red light signals. Thus the observed requirement for RsbP could simply reflect its critical role in countering the RsbW kinase. We note that the σ^B^ signaling network of *Listeria monocytogenes* is also activated by red light [40], yet this bacterium lacks both the RsbQ hydrolase and the RsbP phosphatase [41]. Either two very different red light sensing systems regulate σ^B^ activity in the related *B. subtilis* and *L. monocytogenes*, or the RsbQ-PAS module senses a parameter other than red light.

Identification of the substrate for the RsbQ hydrolase would provide an important clue to the signal sensed by the RsbQ-PAS module. Among the bacteria shown in Fig. 3, RsbQ sequences are more highly conserved than the corresponding PAS sequences, particularly in residues thought to be important for RsbQ substrate binding and specificity (Fig. S2). Thus it is likely that all RsbQ homologues act on a similar substrate. Presumably, the RsbQ-substrate interaction as well as the RsbQ-PAS interaction can be adjusted to grade the sensitivity of the module, thus optimizing it to function in different signaling contexts.

## Materials and Methods

### Bacterial strains and genetic methods

All constructed *Bacillus subtilis* strains shown in Table 2 are derivatives of PB2, a 168 Marburg strain originally obtained from Patrick Piggot [42]. Plasmids used for strain constructions are shown in Table 3. Standard recombinant methods and natural transformation of *B. subtilis* were as described by Brody *et al*. [9].

**Table 2:** *Bacillus subtilis* strains.

Strain	Genotype	Reference or construction^a^
PB2	*trpC*2	168 Marburg strain [42]
PB198	*amyE*::*ctc*-*lacZ trpC*2	[48]
PB715	*rsbP*Δ2 *amyE*::*ctc*-*lacZ trpC*2	K. Vijay, PhD thesis UC Davis 2001
PB1058	*rsbP*C10A *rsbP*Δ2 *amyE*::*ctc*-*lacZ trpC*2	pNE5960 → PB715
PB1059	*rsbP*L13A *rsbP*Δ2 *amyE*::*ctc*-*lacZ trpC*2	pNE5961 → PB715
PB1060	*rsbP*L48A *rsbP*Δ2 *amyE*::*ctc*-*lacZ trpC*2	pNE5962 → PB715
PB1061	*rsbP*Q53A *rsbP*Δ2 *amyE*::*ctc*-*lacZ trpC*2	pNE5963 → PB715
PB1062	*rsbP*F55A *rsbP*Δ2 *amyE*::*ctc*-*lacZ trpC*2	pNE5964 → PB715
PB1063	*rsbP*C56A *rsbP*Δ2 *amyE*::*ctc*-*lacZ trpC*2	pNE5965 → PB715
PB1064	*rsbP*Y59A *rsbP*Δ2 *amyE*::*ctc*-*lacZ trpC*2	pNE5966 → PB715
PB1065	*rsbP*E72A *rsbP*Δ2 *amyE*::*ctc*-*lacZ trpC*2	pNE5967 → PB715
PB1067	*rsbP*P86K *rsbP*Δ2 *amyE*::*ctc*-*lacZ trpC*2	pNE5969 → PB715

a Arrow indicates transformation from donor to recipient.

**Table 3:** Plasmids used for strain construction.

Plasmid	Alteration or relevant feature	Reference
pUS19	Integrative plasmid	[49]
pNE5959	1.8 kb fragment (containing the 3′ 445 bp of *rsbQ*, all of *rsbP,* and an additional 110 bp downstream) cloned into the *Eco*RI site of pUS19	This study
pNE5960	*rsbP*C10A in pNE5959 (TGC→GCC)	This study
pNE5961	*rsbP*L13A in pNE5959 (CTC→GCC)	This study
pNE5962	*rsbP*L48A in pNE5959 (TTG→GCG)	This study
pNE5963	*rsbP*Q53A in pNE5959 (CAG→GCG)	This study
pNE5964	*rsbP*F55A in pNE5959 (TTC→GCC	This study
pNE5965	rsbPC56A in pNE5959 (TGC→GCC)	This study
pNE5966	*rsbP*Y59A in pNE5959 (TAT→GCT)	This study
pNE5967	*rsbP*E72A in pNE5959 (GAA→GCA)	This study
pNE5969	*rsbP*P86A in pNE5959 (CCC→GCC)	This study

Site-directed mutagenesis of *rsbP* was done using the integrative plasmid pNE5959 as a template. pNE5959 carried 445 bp from the 3′ portion of the *rsbQ* coding sequence, the entire *rsbP* coding sequence, and an additional 110 bp downstream from the *rsbP* termination codon. It was generated by amplifying a 1.8 kb fragment from *B. subtilis* genomic DNA with primers QP_*Eco*RI (5′-TACCGGAATTCCTTGCTATTTGAATGATCCG-3′) and P_*Eco*RI_Rev_full (5′- TACCGGAATTCCTTCCTTGCAGGTGTTTCA-3′), both containing *Eco*RI sites (underlined). The resulting fragment was cloned into *Eco*RI-cut pUS19 to yield pNE5959. Point mutations were made in the *rsbP* coding sequence using the QuikChange Lightning kit according to the manufacturer's protocol (Stratagene, La Jolla, CA), creating alanine substitutions at C10, L13, L48, Q53, F55, C56, Y59, E72, and P86. The expected alteration and the integrity of the *rsbP* coding sequence in each construct were confirmed by DNA sequencing.

These substitution constructs were transformed into strain PB715 (*rsbP*Δ2 *amyE*::*ctc-lacZ*). In addition to the *rsbP*Δ2 in-frame deletion, which removed triplets 31–117 from the *rsbP* coding sequence, PB715 also contains a σ^B^-dependent *ctc-lacZ* reporter fusion in single copy at the *amyE* locus. The resulting transformants were merodiploids with only one copy of *rsbP* expressed from the *rsbQP* promoter. Transformants with the active copy containing the RsbP substitutions (and the inactive copy containing the *rsbP*Δ2 deletion) were identified by PCR screen.

### β-galactosidase accumulation assays

Bacterial shake cultures were grown at 37°C in Buffered Luria Broth medium lacking salt (BLB medium [43]). After reaching mid-exponential phase, cells were diluted 1∶25 into fresh BLB and allowed to grow into stationary phase, eliciting the energy stress response. Samples were collected at the times indicated and treated according to Miller [44], as previously described [9]. Activity was defined as ΔA_420_×1,000 per minute per mg of protein (measured with Protein Assay Reagent; Bio-Rad Laboratories, Hercules, CA).

### Detection of RsbP and its mutant variants by Western blotting

To compare steady-state levels of RsbP and its mutant forms, cells were harvested one generation (20 min) before onset of stationary phase and broken by sonication. Total cell protein was separated by SDS-PAGE, transferred to polyvinylidene difluoride membranes (Bio-Rad Laboratories), and probed with rabbit anti-RsbP antibody, as previously described [13]. After exposure to primary antibody, membranes were washed and incubated with IgG peroxide-conjugated anti-rabbit secondary antibody (Santa Cruz Biotechnology Inc., Santa Cruz, CA). Bound antibody was detected using the ECL Plus Western Blotting kit (GE Healthcare Bio-Sciences, Piscataway, NJ) according to the manufacturer's instructions. Images were captured on X-ray film and quantified using an Epson V300 scanner (Epson America, Long Beach CA) and ImageQuant 5.2 software (GE Healthcare Bio-Sciences, Piscataway NJ).

### Yeast two-hybrid methods

We used the Matchmaker Two-Hybrid System (Clontech Laboratories, Mountain View, CA) to compare interactions between RsbQ and wild or mutant versions of RsbP. Full-length *rsbQ* and *rsbP* reading frames were previously fused to either the GAL4 DNA binding domain in plasmid pGBT9 or the GAL4 activation domain in plasmid pGAD424 [9]. Here we made similar constructs expressing fusions to full-length *rsbP* reading frames encoding PAS null substitutions L48A, F55A, Y59A, E72A, and P86A. We also made constructs expressing fusions to different regions of *rsbP*: PAS alone (residues 1-111); predicted coiled-coil and PPM phosphatase (110–403); or PPM alone (167–403). Integrity of the fusion junctions and coding regions was confirmed by sequencing. Constructs were then paired in yeast SFY526 cells by selecting double transformants on minimal plates. Transcriptional activation of the *lacZ* reporter gene in the resulting transformants was determined qualitatively using a colony lift filter assay, or quantitatively by performing β-galactosidase assays on cultures grown in liquid media, according to the manufacturer's protocol. Activities of the RsbP, RsbP-F55A, or RsbP-P86A constructs in the GAL4 DNA binding domain alone (self-activation) were subtracted from the pairwise activities shown in Fig. 5B.

### Assays using purified RsbP

Plasmids encoding His-tagged wild or the F55A mutant form of RsbP in the Novagen pET15b vector (EMD Chemicals, Gibbstown NJ) were transformed into *E. coli* BL21 (DE3). Expression was induced by IPTG and proteins purified under native conditions on nickel affinity columns (Qiagen Inc., Valencia CA), essentially according to the manufacturer's protocol. Following the standard wash with buffer NPI-20 (50 mM NaH_2_PO_4_, pH 8, 300 mM NaCl, 20 mM imidazole), we employed two initial step gradients of NPI-50 and NPI-100 (containing 50 mM and 100 mM imidazole, respectively). RsbP or RsbP-F55A was then eluted with NPI-250 (containing 250 mM imidazole). Concentrations were determined with the Protein Assay Reagent, and purity was assessed by SDS-PAGE.

For pull-down assays, fractions containing His-tagged wild or mutant RsbP were desalted using Zeba Desalt Spin Columns (Thermo Scientific, Rockford IL) and stored in 20 mM Tris-HCl, pH 6.9. 1 µg wild or mutant RsbP was bound to 10 µl of Ni-NTA magnetic agarose beads (Qiagen) in a total volume of 25 µl Buffer A (50 mM NaH_2_PO_4_, pH 8, 50 mM NaCl, 20 mM imidazole). The RsbP-coated beads were added to clarified extract from sonicated, stationary-phase *B. subtilis* cells (300 µg protein in 100 µl Buffer A); this mixture was incubated for 1 hour at 37°C on a roller drum. The supernatant was removed, the beads were washed three times with 100 µl aliquots of Buffer A, and RsbP was eluted with Buffer B (50 mM NaH_2_PO_4_, pH 8, 50 mM NaCl, 250 mM imidazole, 0.05% Tween 20). Supernatant (unbound) and elution (bound) fractions were separated by SDS-PAGE; Western blots were probed with anti-RsbQ antibody [9]. Because this antibody was raised using purified, His-tagged RsbQ as antigen, it recognized both wild type RsbQ in cell extracts and the His-tagged RsbP eluted from the beads. Control pull-down reactions used (i) beads coated with *E. coli* proteins from the same affinity column fraction in which RsbP would normally elute, but isolated from BL21 (DE3) cells containing an empty pET15b vector; and (ii) untreated beads alone.

For size exclusion chromatography, fractions containing wild or mutant RsbP were concentrated to 2.5 mg/ml in Buffer C (20 mM Tris-HCl, pH 6.9, 100 mM NaCl, 0.1 mM EDTA) using Amicon Centricon-30 filters (EMD Millipore, Billerica MA), then applied to a Superose 12 column (10/300 GL; Amersham Biosciences, Piscataway NJ), equilibrated at 4°C with the same buffer. Proteins were eluted at a flow rate of 0.25 ml/min, using a Bio-Rad automated fast protein liquid chromatography system. Column calibration was done with a kit of standard proteins (Sigma-Aldrich, St. Louis MO).

### Bioinformatic methods and computational modeling

Analysis of bacterial genomes for the presence of RsbP-PAS homologues was done using the PSI-BLAST algorithm [17] at the NCBI, with default settings. RsbP residues 1–109 comprise the PAS domain [13] and were used as the first iteration query against the non-redundant database (February 10, 2009 version). From the resulting hits we manually selected those whose structural genes were adjacent to a clear *rsbQ* homologue, based on the Reference Sequence Database [45]. This selected set was then used to build the PSSM matrix for the second PSI-BLAST iteration. The 44 highest-scoring PAS sequences returned from the second iteration all possessed an adjacent *rsbQ* homologue; these PAS sequences formed the core of the dataset for further analysis. One additional sequence not clustered with the first 44 was found manually (*Alteromonas macleodii)*. It was added to the dataset for a total of 45 RsbP-PAS-like sequences.

Multiple alignment of the 45-member dataset was done with AlignX from the Vector NTI 9.0.0 software package (Invitrogen Life Science Software, Carlsbad, CA), using the BLOSUM62 scoring matrix and default program settings; the dendogram shown in Fig. 2 was generated from this alignment using the neighbor-joining method [46]. Domain architecture of PAS-containing proteins was analyzed using the Conserved Domain Database [19]. Probability of sequences forming parallel coiled-coils was evaluated with the pcoil program run with the MTIDK matrix and a weighting for non-polar residues at appropriate positions, using a scanning window of 21 residues [47]. Probability of 0.5 or greater over at least 21 residues was taken as a positive result.

Multiple alignment of the 24 most diverse sequences from Fig. 2 was done with ClustalW [20] employing default settings and the BLOSUM 62 matrix. These 24 sequences were identified by the HHfilter utility from the MPI Bioinformatics Toolkit [21] (http://toolkit.tuebingen.mpg.de/hhfilter). The alignment of the 24 representative RsbP-PAS-like sequences shown in Fig. 3A follows the standard ClustalW color code for amino acid residues, as described in the figure legend. For comparison we performed a similar multiple alignment of a set of outgroup sequences that are closely related to RsbP-PAS but lack an RsbQ partner. From the same PSI-BLAST search that provided the 45 RsbP-PAS-like sequences shown in Fig. 2, we chose the 45 next most closely related as the outgroup. Within this set the 24 most diverse sequences were identified by HHfilter; these were aligned against the previous profile of the 24 RsbP-PAS-like sequences using ClustalW (Fig. S1).

Logos shown in Fig. 3B were generated by the weblogo [24] server (http://weblogo.berkeley.edu/) and represent the relative conservation of the predicted Fα helices of RsbP-PAS-like and outgroup sequences. Tertiary structure of the RsbP PAS domain shown in Fig. 3C was modeled by the i-tasser server (http://zhanglab.ccmb.med.umich.edu/I-TASSER/); this model had a favorable confidence score of 0.80, suggesting an RMSD of 2.6 (+/- 1.9) Å, relative to the true structure [26]. RsbP residues 1-109 were threaded onto the structures of four PAS domains the server chose as the best templates: *Sinorhizobium meliloti* FixL (PDB ID: 1EW0_A), *B. subtilis* YtvA (PDB ID: 2PR5), *E. coli* DosH (PDB ID: 1S66_L), and *Azotobacter vinelandii* NifL (PDB ID: 2GJ3_A). These templates all have bound heme or flavin cofactors. However, thus far no cofactor has been found associated with *B. subtilis* RsbP (MSB and CWP, unpublished).

## Supporting Information

Figure S1 **Multiple alignment of PAS homologues from the comparison outgroup.** 24 representative sequences related to RsbP-PAS but missing a clear RsbQ partner were aligned to the ClustalW profile of RsbP-PAS-like domains that have an RsbQ partner (Fig. 3A). Sequences are labeled by genus-species abbreviation and UniProt identifier [18]. Color scheme is blue for WLVIMFAC; cyan for HY; green for TSNQ; magenta for DE; red for KR; orange for G; and yellow for P. Black circles indicate positions corresponding to the nine residues chosen for analysis in *B. subtilis* RsbP (Fig. 3A). The plot below the alignment shows average sequence conservation. Most members of the comparison outgroup terminate with the DIT motif, which is thought to couple signaling changes within the PAS domain to a succeeding α helix or coiled coil [3], [22], [23]. This motif is absent from RsbP-PAS-like domains that form RsbQ-PAS modules (Fig. 3A).
(TIF)

Figure S2 **Multiple alignment of RsbQ homologues.** ClustalW alignment of the 24 RsbQ homologues encoded immediately adjacent to the RsbP-PAS-like domains shown in Fig. 3A. RsbQ sequences share greater identity than RsbP-PAS sequences, with RsbQ having an average of 48% identity with its homologues (this figure) compared with the 27% average for RsbP-PAS (Fig. 3A). RsbQ sequences are labeled by genus-species abbreviation and UniProt identifier. Numbers on the right indicate the terminal residue in that row. Color scheme is as described in Fig S1 legend, but here C is shown in pink when more than 85% conserved. The structural analysis of Kaneko *et al*. [16] identified the catalytic triad, indicated by the red diamonds above S96, D219 and H247 of *B. subtilis* RsbQ (Bsu_O07015), and these residues are highly conserved. The same analysis suggested additional residues that could affect RsbQ function, indicated by the black circles above F27, V97, F136 and F196, and these too are highly conserved. F27 and V97 were proposed to interact with substrate in the hydrophobic cavity that contains the catalytic triad; F136 and particularly F196 were proposed to impede substrate access to the same cavity. This cavity and a unique loop adjacent to F136 distinguish RsbQ from similar haloperoxidases. The position of this loop is shown by a black line above L126-G135; loop length is variable but its N- and C-terminal regions are conserved. Kaneko *et al*. [16] speculated that the loop provides a site of direct contact between RsbQ and RsbP, and may influence the gating of the hydrophobic cavity of RsbQ to control delivery of a small molecule to its partner.
(TIF)

## References

[1] Pawson T, Nash P (2003). Assembly of cell regulatory systems through protein interaction domains.. Science.

[2] Taylor BL, Zhulin IB (1999). PAS domains: internal sensors of oxygen, redox potential, and light.. Microbiol Mol Biol Rev.

[3] Möglich A, Ayers RA, Moffat K (2009). Structure and signaling mechanism of Per-ARNT-Sim domains.. Structure.

[4] Zhou YF, Nan B, Nan J, Ma Q, Panjikar S (2008). C4-dicarboxylates sensing mechanism revealed by the crystal structures of DctB sensor domain.. J Mol Biol.

[5] Key J, Hefti M, Purcell EB, Moffat K (2007). Structure of the redox sensor domain of *Azotobacter vinelandii* NifL at atomic resolution: signaling, dimerization, and mechanism.. Biochemistry.

[6] Möglich A, Moffat K (2007). Structural basis for light-dependent signaling in the dimeric LOV domain of the photosensor YtvA.. J Mol Biol.

[7] Park H, Suquet C, Satterlee JD, Kang C (2004). Insights into signal transduction involving PAS domain oxygen-sensing heme proteins from the X-ray crystal structure of *Escherichia coli* Dos heme domain (*Ec* DosH).. Biochemistry.

[8] Kurokawa H, Lee DS, Watanabe M, Sagami I, Mikami B (2004). A redox-controlled molecular switch revealed by the crystal structure of a bacterial heme PAS sensor.. J Biol Chem.

[9] Brody MS, Vijay K, Price CW (2001). Catalytic function of an α/β hydrolase is required for energy stress activation of the σ^B^ transcription factor in *Bacillus subtilis*.. J Bacteriol.

[10] Vijay K, Brody MS, Fredlund E, Price CW (2000). A PP2C phosphatase containing a PAS domain is required to convey signals of energy stress to the σ^B^ transcription factor of *Bacillus subtilis*.. Mol Microbiol.

[11] Hecker M, Pané-Farré J, Völker U (2007). SigB-dependent general stress response in *Bacillus subtilis* and related gram-positive bacteria.. Annu Rev Microbiol.

[12] Price CW, Storz G, Hengge R (2010). General stress response in *Bacillus subtilis* and related Gram positive bacteria.. Bacterial stress responses.

[13] Brody MS, Stewart V, Price CW (2009). Bypass suppression analysis maps the signalling pathway within a multidomain protein: the RsbP energy stress phosphatase 2C from *Bacillus subtilis*.. Mol Microbiol.

[14] Bork P, Brown NP, Hegyi H, Schultz J (1996). The protein phosphatase 2C (PP2C) superfamily: detection of bacterial homologues.. Protein Sci.

[15] Zhang W, Shi L (2004). Evolution of the PPM-family protein phosphatases in *Streptomyces*: duplication of catalytic domain and lateral recruitment of additional sensory domains.. Microbiology.

[16] Kaneko T, Tanaka N, Kumasaka T (2005). Crystal structures of RsbQ, a stress-response regulator in *Bacillus subtilis*.. Protein Sci.

[17] Altschul SF, Madden TL, Schaffer AA, Zhang J, Zhang Z (1997). Gapped BLAST and PSI-BLAST: a new generation of protein database search programs.. Nucleic Acids Res.

[18] The UniProt Consortium (2010). The Universal Protein Resource (UniProt) in 2010.. Nucleic Acids Res.

[19] Marchler-Bauer A, Anderson JB, Chitsaz F, Derbyshire MK, DeWeese-Scott C (2009). CDD: specific functional annotation with the Conserved Domain Database.. Nucleic Acids Res.

[20] Thompson JD, Higgins DG, Gibson TJ (1994). CLUSTAL W: improving the sensitivity of progressive multiple sequence alignment through sequence weighting, position-specific gap penalties and weight matrix choice.. Nucleic Acids Res.

[21] Biegert A, Mayer C, Remmert M, Söding J, Lupas AN (2006). The MPI Bioinformatics Toolkit for protein sequence analysis.. Nucleic Acids Res.

[22] Möglich A, Ayers RA, Moffat K (2009). Design and signaling mechanism of light-regulated histidine kinases.. J Mol Biol.

[23] Möglich A, Ayers RA, Moffat K (2010). Addition at the molecular level: signal integration in designed Per-ARNT-Sim receptor proteins.. J Mol Biol.

[24] Crooks GE, Hon G, Chandonia JM, Brenner SE (2004). WebLogo: a sequence logo generator.. Genome Res.

[25] Makino M, Kondo S, Kaneko T, Baba S, Hirata K (2009). Expression, crystallization and preliminary crystallographic analysis of the PAS domain of RsbP, a stress-response phosphatase from *Bacillus subtilis*.. Acta Crystallogr Sect F Struct Biol Cryst Commun.

[26] Roy A, Kucukural A, Zhang Y (2010). I-TASSER: a unified platform for automated protein structure and function prediction.. Nat Protoc.

[27] Antelmann H, Zuber P, Spiro S, Dixon R (2010). Thiol-based sensory factors.. Sensory mechanisms in bacteria: molecular aspects of signal recognition.

[28] Miyatake H, Mukai M, Park SY, Adachi S, Tamura K (2000). Sensory mechanism of oxygen sensor FixL from *Rhizobium meliloti*: crystallographic, mutagenesis and resonance Raman spectroscopic studies.. J Mol Biol.

[29] Serebriiskii IG, Golemis EA (2000). Uses of *lacZ* to study gene function: evaluation of β-galactosidase assays employed in the yeast two-hybrid system.. Anal Biochem.

[30] Neiditch MB, Federle MJ, Pompeani AJ, Kelly RC, Swem DL (2006). Ligand-induced asymmetry in histidine sensor kinase complex regulates quorum sensing.. Cell.

[31] Lee J, Tomchick DR, Brautigam CA, Machius M, Kort R (2008). Changes at the KinA PAS-A dimerization interface influence histidine kinase function.. Biochemistry.

[32] Neiditch MB, Federle MJ, Miller ST, Bassler BL, Hughson FM (2005). Regulation of LuxPQ receptor activity by the quorum-sensing signal autoinducer-2.. Mol Cell.

[33] Schirmer T, Jenal U (2009). Structural and mechanistic determinants of c-di-GMP signalling.. Nat Rev Microbiol.

[34] Szurmant H, White RA, Hoch JA (2007). Sensor complexes regulating two-component signal transduction.. Curr Opin Struct Biol.

[35] Alper S, Dufour A, Garsin DA, Duncan L, Losick R (1996). Role of adenosine nucleotides in the regulation of a stress-response transcription factor in *Bacillus subtilis*.. J Mol Biol.

[36] Delumeau O, Lewis RJ, Yudkin MD (2002). Protein-protein interactions that regulate the energy stress activation of σ^B^ in *Bacillus subtilis*.. J Bacteriol.

[37] Eymann C, Hecker M (2001). Induction of σ^B^-dependent general stress genes by amino acid starvation in a *spo0H* mutant of *Bacillus subtilis*.. FEMS Microbiol Lett.

[38] Zhang S, Haldenwang WG (2005). Contributions of ATP, GTP, and redox state to nutritional stress activation of the *Bacillus subtilis* σ^B^ transcription factor.. J Bacteriol.

[39] Ávila-Pérez M, van der Steen JB, Kort R, Hellingwerf KJ (2010). Red light activates the σ^B^-mediated general stress response of *Bacillus subtilis* via the energy branch of the upstream signaling cascade.. J Bacteriol.

[40] Ondrusch N, Kreft J (2011). Blue and red light modulates SigB-dependent gene transcription, swimming motility and invasiveness in *Listeria monocytogenes*.. PLoS One.

[41] Glaser P, Frangeul L, Buchrieser C, Rusniok C, Amend A (2001). Comparative genomics of *Listeria* species.. Science.

[42] Piggot PJ (1973). Mapping of asporogenous mutations of *Bacillus subtilis*: a minimum estimate of the number of sporeulation operons.. J Bacteriol.

[43] Boylan SA, Redfield AR, Brody MS, Price CW (1993). Stress-induced activation of the σ^B^ transcription factor of *Bacillus subtilis*.. J Bacteriol.

[44] Miller JH (1972). Experiments in Molecular Genetics..

[45] Pruitt KD, Tatusova T, Maglott DR (2007). NCBI reference sequences (RefSeq): a curated non-redundant sequence database of genomes, transcripts and proteins.. Nucleic Acids Res.

[46] Saitou N, Nei M (1987). The neighbor-joining method: a new method for reconstructing phylogenetic trees.. Mol Biol Evol.

[47] Gruber M, Söding J, Lupas AN (2006). Comparative analysis of coiled-coil prediction methods.. J Struct Biol.

[48] Boylan SA, Rutherford A, Thomas SM, Price CW (1992). Activation of *Bacillus subtilis* transcription factor σ^B^ by a regulatory pathway responsive to stationary-phase signals.. J Bacteriol.

[49] Benson AK, Haldenwang WG (1993). Regulation of σ^B^ levels and activity in *Bacillus subtilis*.. J Bacteriol.

