# Supplementary figures and images for: An α/β Hydrolase and Associated Per-ARNT-Sim Domain Comprise a Bipartite Sensing Module Coupled with Diverse Output Domains
Source: PLoS One. 2011 Sep 29;6(9):e25418. doi: 10.1371/journal.pone.0025418 (PMC3183031; doi:10.1371/journal.pone.0025418)

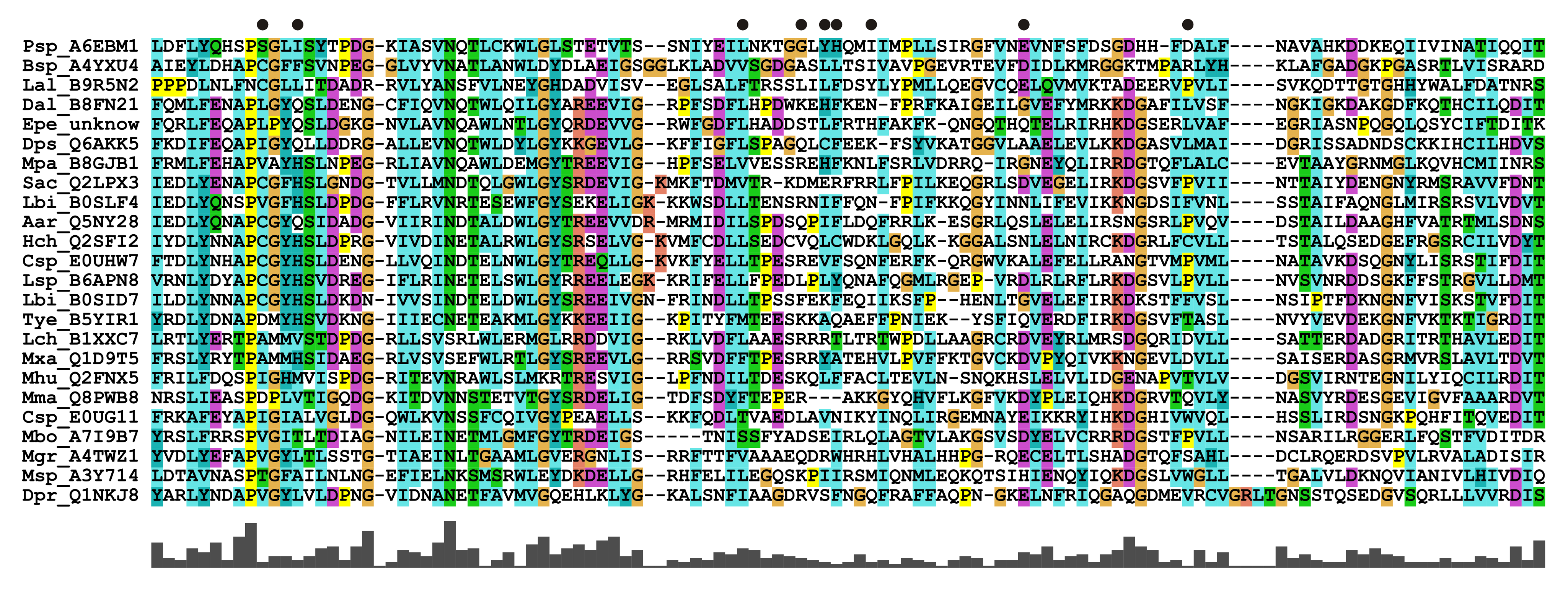

Supplement: Figure S1 — Multiple alignment of PAS homologues from the comparison outgroup. 24 representative sequences related to RsbP-PAS but missing a clear RsbQ partner were aligned to the ClustalW profile of RsbP-PAS-like domains that have an RsbQ partner (Fig. 3A). Sequences are labeled by genus-species abbreviation and UniProt identifier [18]. Color scheme is blue for WLVIMFAC; cyan for HY; green for TSNQ; magenta for DE; red for KR; orange for G; and yellow for P. Black circles indicate positions corresponding to the nine residues chosen for analysis in B. subtilis RsbP (Fig. 3A). The plot below the alignment shows average sequence conservation. Most members of the comparison outgroup terminate with the DIT motif, which is thought to couple signaling changes within the PAS domain to a succeeding α helix or coiled coil [3], [22], [23]. This motif is absent from RsbP-PAS-like domains that form RsbQ-PAS modules (Fig. 3A). (TIF) [file pone.0025418.s001.tif]

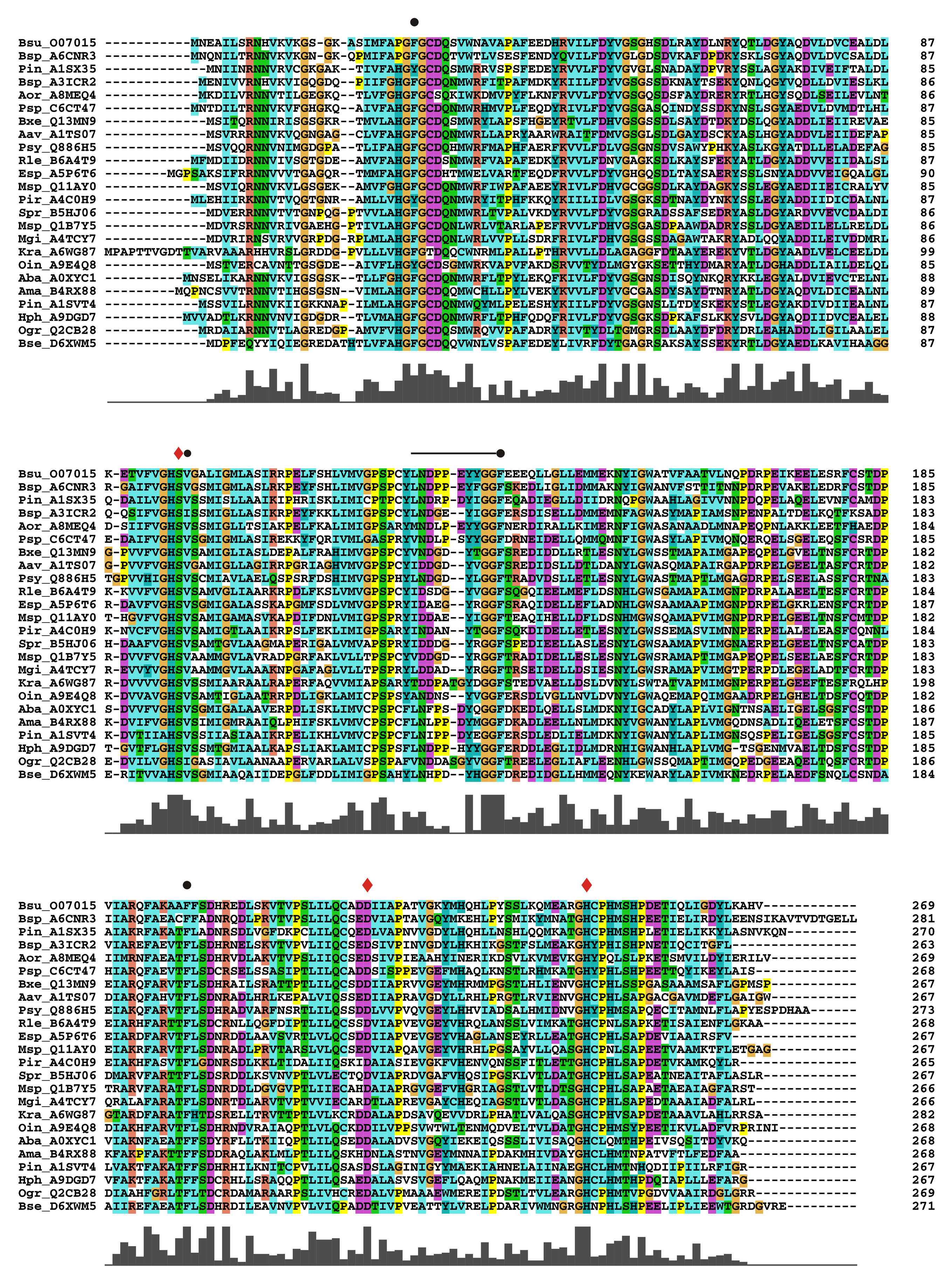

Supplement: Figure S2 — Multiple alignment of RsbQ homologues. ClustalW alignment of the 24 RsbQ homologues encoded immediately adjacent to the RsbP-PAS-like domains shown in Fig. 3A. RsbQ sequences share greater identity than RsbP-PAS sequences, with RsbQ having an average of 48% identity with its homologues (this figure) compared with the 27% average for RsbP-PAS (Fig. 3A). RsbQ sequences are labeled by genus-species abbreviation and UniProt identifier. Numbers on the right indicate the terminal residue in that row. Color scheme is as described in Fig S1 legend, but here C is shown in pink when more than 85% conserved. The structural analysis of Kaneko et al. [16] identified the catalytic triad, indicated by the red diamonds above S96, D219 and H247 of B. subtilis RsbQ (Bsu_O07015), and these residues are highly conserved. The same analysis suggested additional residues that could affect RsbQ function, indicated by the black circles above F27, V97, F136 and F196, and these too are highly conserved. F27 and V97 were proposed to interact with substrate in the hydrophobic cavity that contains the catalytic triad; F136 and particularly F196 were proposed to impede substrate access to the same cavity. This cavity and a unique loop adjacent to F136 distinguish RsbQ from similar haloperoxidases. The position of this loop is shown by a black line above L126-G135; loop length is variable but its N- and C-terminal regions are conserved. Kaneko et al. [16] speculated that the loop provides a site of direct contact between RsbQ and RsbP, and may influence the gating of the hydrophobic cavity of RsbQ to control delivery of a small molecule to its partner. (TIF) [file pone.0025418.s002.tif]
